# Molecular Mechanisms of NF-Y Transcription Factors in Horticultural Plant Development and Stress Responses: Recent Advances

**DOI:** 10.3390/ijms27031443

**Published:** 2026-01-31

**Authors:** Mengxia Zhang, Dan Chen, Chunjuan Dong

**Affiliations:** 1 State Key Laboratory of Vegetable Biobreeding, Institute of Vegetables and Flowers, Chinese Academy of Agricultural Sciences, Beijing 100081, China; 2 Hainan Institute, Zhejiang University, Sanya 572025, China; chendan137@zju.edu.cn

**Keywords:** NF-Y transcription factors, horticultural plants, developmental regulation, stress response, molecular mechanism, CCAAT cis-element, gene regulatory network

## Abstract

Nuclear Factor Y (NF-Y) transcription factors are evolutionarily conserved regulators that bind the CCAAT box, playing central roles in horticultural plant growth and adaptation. This review summarizes recent progress on NF-Ys in horticultural plants, focusing on their molecular mechanisms in development and stress responses. For development, NF-Ys mediate phase transition, flowering regulation, embryogenesis, and organ development by integrating endogenous signals (gibberellic acid, GA; abscisic acid, ABA) and regulating downstream genes. For stress responses, they enhance tolerance to abiotic stresses (drought, salt, extreme temperatures) via regulating reactive oxygen species (ROS) scavenging, ABA biosynthesis, and stress networks, and mediate biotic stress resistance (e.g., pathogen infection) by activating defense pathways. This review also briefly covers species-specific genomic features (e.g., duplication-driven expansion) and structural traits (conserved core domains, variable termini) underpinning NF-Y specialization. Finally, it highlights key knowledge gaps (e.g., incomplete regulatory networks, limited translational application) and proposes future directions: deciphering NF-Y crosstalk, exploring combined stress responses, accelerating functional validation of uncharacterized *NF-Y* genes, and translating research into horticultural breeding. This work provides a holistic reference for understanding NF-Y function and improving horticultural plant yield, quality, and stress resilience.

## 1. Introduction

Horticultural plants, encompassing fruits, vegetables, ornamental flowers, and medicinal herbs, are vital components of global food security, economic sustainability, and ecological balance. They contribute significantly to human nutrition, aesthetic value, and agricultural diversification, while facing relentless challenges from abiotic stresses (e.g., drought, salt, extreme temperatures) and biotic stresses (e.g., pathogen infections) amid global climate change. Additionally, precise regulation of developmental processes—such as phase transition, flowering, seed maturation, and fruit ripening—is critical for optimizing crop yield, quality, and commercial value. Transcription factors (TFs) are key regulatory proteins that orchestrate gene expression networks, integrating environmental cues and endogenous signals to govern plant development and stress adaptation.

Among diverse TF families, NF-Y is a conserved heterotrimeric complex (NF-YA/NF-YB/NF-YC) that specifically binds to the CCAAT cis-element in target gene promoters to modulate transcriptional initiation [1,2,3]. Initially characterized in yeast and animals, NF-Ys have been extensively studied in model plants for their pleiotropic roles in development and stress tolerance [4,5,6]; with advances in high-throughput genome sequencing and functional genomics, NF-Y family members have since been systematically identified in an increasing number of horticultural plants, revealing key species-specific traits—including variations in subunit gene numbers, uneven chromosome distribution, and family expansion driven by gene duplication events [7,8,9]—as well as structural features: conserved core domains ensure trimer assembly and DNA-binding specificity, while variable N/C termini enable species-specific regulatory functions [10,11,12].

Horticultural plants possess a unique set of agronomically vital traits—such as fruit ripening, tuber/bulb organogenesis, ornamental flower development, and perennial growth cycles—that are not central to model plant studies yet are primary targets for yield and quality improvement. Despite substantial progress in dissecting NF-Y functions in individual horticultural species (e.g., tomato, apple, cucumber) [13,14,15,16], a comprehensive synthesis of their molecular mechanisms underlying these and other horticulture-specific processes as well as stress responses across diverse horticultural taxa remains lacking. Moreover, the crosstalk between NF-Y-mediated pathways and other regulatory networks (e.g., hormone signaling, miRNA regulation, epigenetic modifications) is not fully elucidated [17,18,19,20], and the translational potential of NF-Y factors for horticultural crop improvement is yet to be fully exploited. Currently, research on NF-Y in ornamental and medicinal plants remains fragmented. Many species have only undergone genome-wide identification of *NF-Y* genes, with functional characterization still lacking. This represents a significant knowledge gap and a promising direction for future research, particularly in exploring species-specific adaptation mechanisms and secondary metabolic regulation. In this review, we systematically summarize recent advances in the genomic organization, structural features, and molecular mechanisms of NF-Y TFs in horticultural plant development (phase transition, flowering, organogenesis) and stress responses (abiotic and biotic stresses) [1,13,14,15,21,22]. We also highlight knowledge gaps and propose future research directions, aiming to provide a holistic overview for researchers in this field and facilitate the application of NF-Y-related biotechnology in horticultural crop breeding.

This review systematically evaluates studies that advance our mechanistic understanding of NF-Ys in horticultural plants. While prioritizing recent findings from the past five years, it also incorporates foundational earlier work that remains critical to the field. We systematically searched databases including Web of Science, Scopus, PubMed, and Google Scholar using keywords such as “NF-Y”, “horticultural plants”, “stress response”, “development”, and relevant species names. The literature screening prioritized functional studies focusing on the roles of NF-Y in horticultural plant development and stress adaptation, ensuring the relevance and reliability of the included research.

## 2. Genomic Organization and Structural Features of NF-Y Families in Horticultural Plants

NF-Y transcription factors are conserved heterotrimeric complexes (NF-YA/NF-YB/NF-YC) that regulate horticultural plant growth, development, and stress responses [1,2,3]. With genome sequencing advances, their genomic organization and structural traits have been widely studied, revealing species-specific diversity.

### 2.1. Genomic Organization of NF-Y Families in Horticultural Plants

The genomic organization of the NF-Y family in horticultural plants exhibits distinct characteristics, primarily reflected in variations in subunit gene numbers, uneven chromosome distribution, and expansion driven by gene duplication events.

Regarding subunit gene numbers, significant interspecific differences exist, largely shaped by evolutionary processes such as whole-genome duplication (WGD) [3]. For instance, tomato (*Solanum lycopersicum*) harbors a large *NF-Y* family with 10 *NF-YA*, 29 *NF-YB*, and 20 *NF-YC* genes [15,16], while butterfly orchid (*Phalaenopsis* sp.) has a smaller family (4 *NF-YA*, 9 *NF-YB*, 11 *NF-YC*) [23]. Apple (*Malus domestica*), which has experienced multiple WGD events, contains 11 *NF-YA*, 22 *NF-YB*, and 10 *NF-YC* genes [13], highlighting duplication-mediated family expansion that supports adaptive functional differentiation.

*NF-Y* genes are unevenly distributed across chromosomes, with non-random patterns often linked to functional clustering. Cucumber (*Cucumis sativus*) *NF-Y* genes are present on all chromosomes except chromosome 2, with the highest density on chromosomes 3 and 6 (7 genes each) [14]; Pepper (*Capsicum annuum*) has 19 *NF-YB* genes on 7 of its 12 chromosomes, including tandem duplications on chromosome 7 [22]; and Japanese apricot (*Prunus mume*) *NF-Y* genes concentrate on chromosomes 01 and 03 (25.92% each) but are rare on chromosome 06 (3.70%) [24].

Gene duplication—encompassing WGD, segmental duplication, and tandem duplication—is a key driver of *NF-Y* family expansion. The large *NF-Y* family of banana (*Musa acuminata*), consisting of 14 *NF-YA*, 16 *NF-YB*, and 14 *NF-YC* subunits, is primarily driven by WGD [25]. For woodland strawberry (*Fragaria vesca*), segmental duplication underpins the distribution of *NF-Y* genes across its 7 chromosomes [21]. In pepper, tandem duplication leads to the clustering of *NF-YB* genes on chromosome 7 [22]. These duplication events facilitate functional differentiation of *NF-Y* genes, thereby enhancing plants’ adaptability to various stresses.

Detailed genomic organization data of NF-Y families and their key functional roles studied across various horticultural plants are summarized in Table 1.

### 2.2. Structural Features of NF-Y Subunits in Horticultural Plants

The function of the NF-Y trimer relies on conserved domains in each subunit. NF-YA contains a CCAAT-binding domain (CBF) that enables specific binding to target gene promoters [1,10], while its N/C termini interact with the NF-YB/NF-YC heterodimer [50]. NF-YB has a histone fold motif (YB_HMF) mediating dimerization with NF-YC, a structure resembling the histone H2A-H2B complex [10]. NF-YC harbors a Heme Activator Protein 5 (HAP5) superfamily domain that stabilizes NF-YB/NF-YC dimerization via hydrophobic interactions [11].

The trimer assembly follows a strict sequence: NF-YB and NF-YC first form a cytoplasmic heterodimer through their histone fold motifs, translocate to the nucleus, and then bind NF-YA to form a functional trimer [50]. Only the complete trimer binds DNA—NF-YA recognizes the CCAAT box, and the NF-YB/NF-YC heterodimer stabilizes this DNA interaction [10]. Notably, higher plants have retained more NF-Y subunit paralogs during evolution, and the diverse trimeric combinations derived from these paralogs endow NF-Y complexes with functional specificity—supporting their involvement in various plant developmental processes and responses to different stresses (e.g., drought, salt stresses and fungal infections) [2,3,31]. This combinatorial flexibility, built on the conserved assembly sequence, is considered a core mechanism that accounts for the multifaceted roles of NF-Y in plant adaptation and growth [51].

Core domains (e.g., histone fold motifs in NF-YB/NF-YC) are conserved across horticultural species like tomato, apple, and cucumber [13,14,15,16]. In contrast, variable N/C termini support species-specific functions: NF-YA in sweet orange (*Citrus sinensis*) has drought-responsive motifs [35], and NF-Y subunits in banana carry motifs related to fruit ripening [25]. The conserved core domains of NF-Y subunits underpin the universal assembly and DNA-binding specificity of the heterotrimeric complex across horticultural species. In contrast, the divergent N- and C-terminal regions provide the structural basis for functional diversification. This modular architecture—a conserved “engine” coupled with variable “regulatory modules”—enables the evolution of a large repertoire of NF-Y complexes through combinatorial associations of paralogous subunits. Consequently, the functional specificity of a given NF-Y in processes such as drought response or fruit ripening likely depends not only on its expression pattern but also on the precise subunit composition of the trimer, which may determine interactions with specific co-regulators or chromatin contexts.

**Figure 1:**
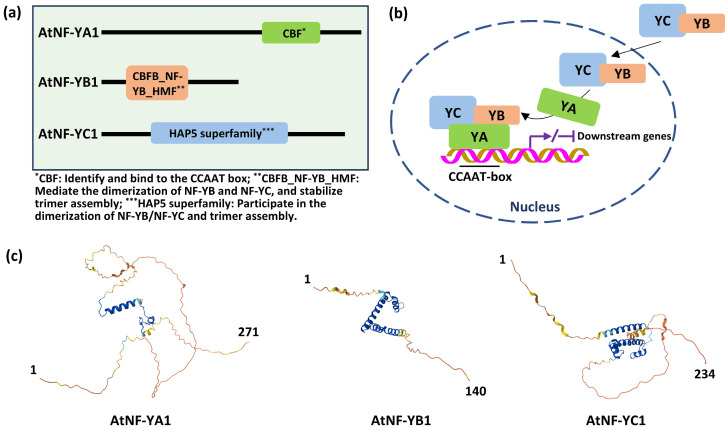
Conserved core structure and assembly mechanism of NF-Y transcription factors, illustrated by the *Arabidopsis* model and exemplified in horticultural plants. (**a**) Domain architectures of *Arabidopsis* NF-Y subunits (AtNF-YA1, AtNF-YB1, and AtNF-YC1). The conserved core domains—CCAAT-binding domain (CBF) in NF-YA, and histone-fold motifs (YB_HMF) in NF-YB/NF-YC—are indispensable for trimer assembly and specific DNA binding. The variable N- and C-terminal regions contribute to functional diversification across horticultural species, enabling species-specific regulatory roles (e.g., fruit ripening in banana [25], drought response in tea plant [27]). (**b**) Schematic of NF-Y trimer-mediated CCAAT-box-dependent transcriptional regulation. The NF-YA/NF-YB/NF-YC heterotrimer translocates to the nucleus and binds the CCAAT cis-element in target gene promoters, thereby modulating downstream gene expression. This core regulatory mechanism is highly conserved across plants and underpins diverse biological functions in horticultural species. For example: In tomato, the SINF-YA3b-containing trimer regulates fruit ripening by binding the promoter of the *SIPS1* gene [54]; In apple, an MdNF-YB18-containing complex interacts with MdNF-YC3/7 to bind the *MdFT1* promoter, promoting flowering transition [55]; In citrus, CsNF-YA5 binds the CCAAT-box of drought-responsive genes, enhancing drought tolerance by regulating antioxidant systems and photosynthesis [35]. Notably, in many of these horticultural examples, the NF-Y complex itself or its target genes are key components of hormone signaling pathways (e.g., ethylene in banana fruit ripening, ABA in tea drought response), positioning NF-Y as a critical integrator of hormonal and environmental signals [25,27]. (**c**) AlphaFold-predicted 3D structures of AtNF-YA1, AtNF-YB1, and AtNF-YC1. The folded conformations of the conserved core domains (highlighted in ribbon structure) are critical for trimer stability and DNA interaction. The overall architectural feature of NF-Y subunits is expected to be conserved in horticultural plant orthologs, supporting the universal application of the *Arabidopsis* model to understand NF-Y function in crops.

Figure 1 takes three typical NF-Y subunits (AtNF-YA1/AtNF-YB1/AtNF-YC1) from *Arabidopsis* as examples to illustrate key structural details of NF-Y subunits—including their domain architectures, the NF-Y trimer-mediated CCAAT-box-dependent transcriptional regulation process, and the AlphaFold-predicted 3D structures of each subunit—all of which underpin the conserved domains and assembly mechanism of NF-Y subunits discussed above [52,53].

## 3. Molecular Mechanisms of NF-Ys in Horticultural Plant Development

Recent studies have elucidated diverse and important roles of NF-Y transcription factors in horticultural plant development. Their functions are particularly critical in two key, interconnected areas: the regulation of phase transition and flowering time (Section 3.1), and the coordination of early embryogenesis, organ morphogenesis, and seed maturation alongside storage compound accumulation (Section 3.2). The underlying molecular mechanisms for these processes are increasingly elucidated. This chapter synthesizes the latest advances in these two pivotal research areas, highlighting how NF-Y complexes integrate diverse signals to direct developmental fate and resource allocation.

### 3.1. Phase Transition and Flowering Control

NF-Y transcription factors function as central integrators within the gene regulatory networks that govern phase transition and flowering time in horticultural plants. They decode a combination of endogenous developmental signals, such as GA and ABA, and environmental cues, including photoperiod and temperature, to precisely regulate the expression of key flowering pathway genes like *FLOWERING LOCUS T* (*FT*) and *SUPPRESSOR OF OVEREXPRESSION OF CONSTANS 1* (*SOC1*). A recurring theme emerging from these studies is the role of NF-Y complexes as integrators of hormone signals—particularly GA and ABA—with environmental and developmental cues to fine-tune flowering time. This section synthesizes recent advances that elucidate how specific NF-Y heterotrimeric complexes—through direct promoter binding, interactions with other transcription factors, and integration with hormonal and epigenetic pathways—orchestrate the precise timing of flowering across diverse horticultural species.

NF-Y transcription factors play pivotal roles in phase transition and flowering control of horticultural plants by mediating target gene expression and signal crosstalk. Li et al. (2025) identified 57 BcNF-Y members in non-heading Chinese cabbage (*Brassica campestris*), and *BcNF-YA8*, upregulated by ABA, promotes flowering via directly activating *BcFT* expression and inhibiting ascorbate accumulation [17]. Conversely, Zhang et al. (2024) reported that tomato SlNF-YA3b negatively regulates flowering by binding to the CCAAT cis-elements of *SFT* (*FT* homolog) promoter, with its knockout inducing early flowering [54]. Pan et al. (2023) demonstrated that lily (*Lilium* spp.) LoNFYA7 recruits *Lilium oriental* VERNALIZATION INSENSITIVE 3-LIKE 1 (LoVIL1)- Polycomb Repressive Complex 2 (PRC2) to enhance H3K27me3 at *Lilium oriental CALLOSE SYNTHASE 3* (*LoCALS3*) locus, repressing callose synthesis and facilitating bud growth transition from dormancy [19]. In summary, NF-Ys exert diverse regulatory effects on phase transition and flowering through distinct molecular pathways. More details about NF-Y transcription factors that regulate phase transition and flowering in horticultural plants are summarized in Table 2.

The collected evidence positions NF-Ys as versatile, context-dependent regulators of flowering, capable of both promotion and repression (Table 2). This functional duality may be resolved by considering three key factors. First, subunit specificity: distinct NF-YA/B/C combinations likely confer unique target gene selectivity. Second, upstream signal integration: the same NF-Y can mediate opposite outcomes depending on whether it is activated by ABA, GA, or other signaling pathways. Third, experimental resolution: while some studies establish direct regulation through promoter binding and genetic interaction, others report correlative evidence. Thus, NF-Ys likely function as integrative nodes within flowering networks, with their output finely tuned by developmental stage, environmental cues, and specific protein partnerships.

### 3.2. Early Embryogenesis, Organ Morphogenesis, and Seed Maturation with Storage Compound Accumulation

Beyond flowering, NF-Y complexes are master regulators of organogenesis and maturation, largely through their capacity to coordinate hormone dynamics (e.g., auxin, ABA, GA, ethylene) and metabolic pathways. They direct the development of agriculturally vital organs and storage tissues by forming heterotrimeric complexes and modulating target gene expression. Specifically, NF-Y transcription factors regulate horticultural plant development—encompassing early embryogenesis, organ morphogenesis, seed maturation, and storage compound accumulation—primarily by forming heterotrimeric complexes and modulating target gene expression, with regulatory roles involving hormone signaling, chloroplast biogenesis, and metabolic pathways. In tomato, SlLEC1-LIKE4 (SlL1L4) harbors a unique N-terminal domain that integrates auxin and ABA signals to coordinate cell division, tissue patterning, and desiccation tolerance critical for early embryogenesis, with ZFN-mediated disruption leading to abnormal embryonic development, altered seed storage compound accumulation and delayed fruit ripening [67]. For cucumber, CsNF-YC2 and CsNF-YC9 directly bind the promoter of *Cucumis sativus TRANSLOCON AT THE INNER MEMBRANE OF CHLOROPLASTS 21* (*CsTIC21*) to enhance its transcription, thereby promoting light-dependent chloroplast photomorphogenesis and leaf development [68]. In potato (*Solanum tuberosum*), the StNF-YA8-YB20-YC5 module activates genes related to GA synthesis and ABA catabolism, altering the ABA/GA balance to accelerate tuber dormancy release, a key process in stolon and tuber organogenesis [69]. For watermelon (*Citrullus lanatus*), ClNF-YB9 interacts with ClNF-YCs and recruits ClNF-YA7 to form a functional trimer, which is critical for embryo morphogenesis and seedling organ formation [33]. In tomato, SlNF-YA8 modulates cotyledon development during embryogenesis and seed maturation, with ZFN-mediated disruption leading to altered seedling establishment, 43% increased fruit weight, rounded fruit shape, and modified locule number that reflect its pleiotropic roles in linking early embryonic development to seed maturation and post-embryonic organ formation [58]. Together, these findings underscore the conserved and diverse roles of NF-Y transcription factors across the sequential processes of early embryogenesis, organ morphogenesis, and seed maturation, with detailed information on related genes and mechanisms summarized in Table 3.

Beyond their ancestral role in embryogenesis, NF-Ys have been co-opted for the regulation of horticulturally vital processes, such as fruit ripening, tuber development, and seed storage compound accumulation. This functional expansion in horticultural species often involves conserved regulators like LEC1 (NF-YB9) but targets distinct downstream genes affecting texture, color, flavor, and nutrition [33,34,70]. The mechanistic studies, particularly in tomato and potato, reveal that NF-Ys interface with hormone (ethylene, ABA) and sugar signaling networks to control these specialized developmental programs. However, the extent to which the underlying regulatory logic is conserved between, for example, fruit ripening and seed maturation, or is newly evolved in specific lineages, remains an open and compelling question.

## 4. Molecular Mechanisms of NF-Ys in Horticultural Plant Stress Responses

Horticultural plants are frequently exposed to diverse abiotic stresses (e.g., drought, salt, extreme temperatures) and biotic stresses (e.g., pathogen infections) during growth and development, which severely affect their yield and quality. As key transcriptional regulators, NF-Y factors play pivotal roles in mediating stress-responsive signaling pathways by binding to CCAAT elements in target promoters and coordinating with other regulatory factors to modulate gene expression. This section systematically summarizes recent advances in the molecular mechanisms underlying NF-Y-mediated stress tolerance in horticultural plants, with a focus on major abiotic and biotic stress types.

### 4.1. Drought Stress

NF-Y transcription factors function as integrative hubs that coordinate multiple hormone signaling pathways to mediate drought adaptation. This integrative function is exemplified by their central role in amplifying ABA signaling—a common hub in drought response—while simultaneously interacting with other hormone pathways such as jasmonate (JA) to orchestrate a comprehensive adaptive output. The drought response mediated by NF-Ys predominantly converges on the amplification of ABA signaling and the enhancement of cellular antioxidant capacity. Drought stress severely restricts the growth, yield, and quality of horticultural plants, with NF- Y transcription factors constituting key components of these pathways. In apple (*Malus hupehensis*), the drought- and ABA-induced NF-YA subunit MhNF-YA3-like enhances drought tolerance by directly binding the promoter of the ABA biosynthesis gene *Malus hupehensis Arabidopsis ALDEHYDE OXIDASE 3* (*MhAAO3*) to activate its expression, and its interaction with *Malus hupehensis MULTICOPY SUPPRESSOR OF IRA1 4-LIKE* (*MhMSI4-like*) further amplifies this effect—overexpression reduces leaf wilting and ROS accumulation, while silencing increases susceptibility [76]. In chrysanthemum (*Chrysanthemum morifolium*), the negatively acting *CmNF-YB8* is downregulated by drought; silencing it upregulates *Chrysanthemum morifolium CBL-INTERACTING PROTEIN KINASE 6* (*CmCIPK6*, involved in stomatal adjustment) and *Chrysanthemum morifolium SHINE 3* (*CmSHN3*, involved in cuticle biosynthesis) to reduce water loss, whereas overexpression exacerbates drought damage [77]. In citrus (*Citrus* spp.), drought-induced CiNF-YA1 forms a complex with CiNF-YB2/CiNF-YC2 to activate *CiFT* (mediating drought-induced flowering), and while overexpression increases drought susceptibility via ROS accumulation, silencing enhances tolerance through improved photosynthesis [78]. These studies demonstrate the diverse roles of NF-Ys in horticultural plant drought responses, with the involved mechanisms—hormone biosynthesis, stomatal adjustment, cuticle development, and flowering crosstalk—being only a subset of their regulatory modes. More NF-Y-mediated drought response mechanisms in horticultural plants are summarized in Table 4.

A conserved theme emerging from studies across species is the central role of NF-Ys in amplifying the ABA signaling pathway to orchestrate drought responses, including stomatal closure and expression of protective genes (e.g., *LEA*s, *HSP*s) [18,86,87]. Functional validation for this role is strong in cases like potato *StNF-YA7* and sweet orange *CsNF-YA5* [18,35,84,85]. However, the landscape of evidence is mixed, with several entries supported primarily by expression correlation. Notably, species-specific adaptations exist alongside this ABA core; for instance, CmNF-YB8 in chrysanthemum regulates cuticle morphology, a mechanism less emphasized in other species [77]. This indicates that while a central ABA-connected module is widely utilized, NF-Y networks also contribute to lineage-specific adaptive traits.

### 4.2. Salt Stress

In response to salt stress, which severely impacts horticultural plant growth, NF-Y transcription factors have been widely shown to regulate gene networks essential for salt tolerance. In grapevine (*Vitis amurensis*), *VaNF-YA6* enhances salt tolerance when overexpressed in grapevine leaves and *Arabidopsis* by upregulating stress-responsive genes (e.g., *VvSOS2*, *VvSOS3*) and improving antioxidant enzyme activities [81]. In tomato, SlNF-YC1 interacts with SlMYB1 to strengthen the transcriptional activation of the glutamate decarboxylase gene *SlGAD1*, promoting γ-aminobutyric acid (GABA) accumulation and ROS scavenging, thereby enhancing saline-alkali tolerance [88]. Overexpression of *CsNF-YC6* from tea plant (*Camellia sinensis*) in *Arabidopsis* (*CsNF-YC6-OE* transgenic lines) resulted in increased seed germination and root length under ABA and GA treatments, as well as improved salt stress tolerance by regulating ABA signaling-related genes and increasing proline content [27]. These findings underscore the diverse mechanisms of NF-Ys in mediating salt stress responses, including gene regulation, metabolite accumulation, and antioxidant defense. For a comprehensive summary of NF-Ys involved in salt stress responses in horticultural plants, refer to Table 5.

NF-Ys enhance salt tolerance through two primary, often interconnected, strategies: ionic homeostasis (e.g., regulating SOS pathway genes as seen in grapevine VaNF-YA6 [81]) and osmotic adjustment (e.g., promoting compatible solute accumulation like GABA in tomato via SINF-YC1 [88]). The relative emphasis on these strategies may reflect ecological adaptation. A critical examination reveals that the evidence for direct transcriptional control of ion transporters by NF-Ys is less prevalent than for their role in osmotic stress signaling. Furthermore, the frequent induction of *NF-YA* genes by salt stress, often via downregulation of *miR169*, presents a conserved regulatory layer, yet the functional significance of specific *miR169*-targeted *NF-YA* paralogs in salt tolerance warrants deeper investigation across species [32,89].

### 4.3. Temperature Stress

#### 4.3.1. Heat Stress

Heat stress significantly impairs horticultural plant growth, with NF-Y transcription factors orchestrating stress responses via complex regulatory networks. In cucumber, CsNFYA1 interacts with *Cucumis sativus* MULTIPROTEIN BRIDGING FACTOR 1c (CsMBF1c) to enhance heat tolerance by stabilizing the photosynthetic system and promoting transcriptional activation of heat-related genes [90]. In cabbage (*Brassica oleracea*), *Brassica oleracea* DNA POLYMERASE II SUBUNIT B3-1 (BoDPB3-1)/NF-YC10 acts as a coactivator of *DEHYDRATION-RESPONSIVE ELEMENT BINDING PROTEIN 2A* (*DREB2A*), and its induced expression under heat stress contributes to thermotolerance, while suppressed expression increases heat sensitivity [91]. In tomato, Sly-NF-YA9/A10 function as negative regulators of heat tolerance; miR169-mediated downregulation of these NF-YAs relieves repression of heat stress-responsive genes, with heat shock factors (HSFs) transcriptionally regulating *MIR169* to form a regulatory loop [20]. These findings highlight NF-Ys’ diverse roles in mediating heat stress responses through protein interactions and miRNA regulation. For a comprehensive summary of NF-Y transcription factors and their specific functions in horticultural plants under heat stress, refer to Table 6.

#### 4.3.2. Cold Stress

Cold stress poses a severe threat to horticultural plants, and NF-Y transcription factors mediate cold tolerance through various regulatory mechanisms. In strawberry (*Fragaria ananassa*), *CBF/NF-Y* (*YZ9*)-overexpressed fruits promote coloring under cold treatment, while *HSF20* (*YZ1*)-overexpressed ones are sensitive to cold/heat, regulating fruit quality traits [73]. In melon (*Cucumis melo*), 25 *CmNF-Y*s (6 *NF-YA*s, 11 *NF-YB*s, 8 *NF-YC*s) are identified, with 12 induced by cold stress, highlighting their key role in cold tolerance [36]. In pepper, CaNF-YC1 interacts with the TIFY family member protein CaTIFY7 to form a module, activating *Capsicum annuum C-REPEAT BINDING FACTOR 1a/b* (*CaCBF1a/b*) expression and enhancing cold tolerance by regulating antioxidant enzymes and stress-responsive genes [29]. These findings reveal NF-Ys’ multifaceted roles in cold signaling and tolerance. For a comprehensive summary of NF-Y transcription factors involved in cold stress responses in horticultural plants, refer to Table 7.

NF-Ys demonstrate remarkably diverse, even opposing, functions in temperature stress responses. In heat stress, they can be positive effectors (e.g., cucumber *CsNFYA1* [90]) or negative regulators released by miR169 (e.g., tomato *Sly-NF-YA9/A10* [20]). In cold stress, they often act as positive regulators within CBF-dependent or -independent pathways. This complexity likely arises from their position as hubs that integrate temperature signals with other environmental and developmental cues. A key insight is that their role cannot be generalized as simply “positive” or “negative”; instead, it is conditional, depending on the specific subunit, interacting partners (e.g., DREB2A, MBF1c), and the physiological process being modulated (e.g., photosynthesis protection vs. growth arrest).

### 4.4. Other Abiotic and Biotic Stresses

NF-Y transcription factors are also implicated in a range of additional abiotic and biotic stresses in horticultural plants. In tomato, under oxidative stress, the CCAAT-binding factor SlNFYA10 negatively regulates ascorbate biosynthesis by targeting the GDP- mannose-3′,5′-epimerase gene *SlGME1* and the GDP-L-galactose phosphorylase gene *SlGGP1* in the D-mannose/L-galactose pathway, thereby increasing sensitivity to oxidative stress [92]. In pigeon pea (*Cajanus cajan*), under aluminum (Al) stress, CcNFYB3 enhances citrate efflux by activating *Cajanus cajan MULTIDRUG AND TOXIC COMPOUND EXTRUSION TRANSPORTER 35* (*CcMATE35*), while the *Cajanus cajan* long noncoding RNA targeting citrate synthase (CcLTCS) upregulates the *Cajanus cajan* citrate synthase gene *CcCS* to promote citrate synthesis; these two modules synergistically improve aluminum tolerance [93]. In watermelon, against stresses from fungal (*Fusarium oxysporum*) and bacterial (*Pseudomonas syringae*) pathogens, multiple *ClNF-Y* genes (e.g., *ClNF-YB8*, *ClNF-YA3*) mediate resistance via hormone-related signaling [31]. In common bean (*Phaseolus vulgaris*), for stress caused by the powdery mildew fungus *Erysiphe diffusa*, *NF-YA3* is identified as a candidate gene associated with oligogenic resistance [94]. These findings broaden the functional scope of NF-Ys in both abiotic and biotic stress adaptation. For a comprehensive summary of NF-Y transcription factors involved in additional stress responses in horticultural plants, refer to Table 8.

NF-Ys are recurrently implicated in biotic stress responses, primarily through association with SA- and JA-mediated defense gene activation. However, a critical gap exists between correlation and mechanistic understanding. For most reported NF-Ys, it is unclear whether they directly bind defense gene promoters or exert effects indirectly by modulating hormone signaling components. The observation of both positive (e.g., CINF-YB8) and negative (e.g., CINF-YA2) regulators within the same species suggests a nuanced, subunit-specific regulatory network that is far from decoded [31]. Moreover, the current literature is strikingly focused on fungal and bacterial pathogens, leaving the role of NF-Ys in antiviral defense or resistance to insect herbivory—critical for horticulture—virtually unexplored.

## 5. Conclusions and Future Directions

**Figure 2:**
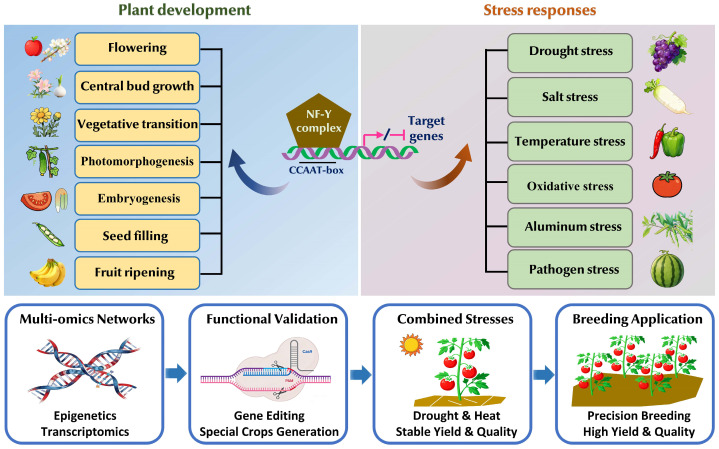
Regulatory roles of NF-Y transcription factors in horticultural plant development and stress responses, and key future research directions.

This review systematically organizes the research progress of NF-Y transcription factors in horticultural plants, with a core focus on their molecular mechanisms in regulating plant developmental processes (e.g., phase transition, flowering, organogenesis) and responding to abiotic/biotic stresses. These core regulatory functions (encompassing representative developmental events and stress types in horticultural species) and the critical future research priorities aimed at addressing existing knowledge gaps are visually synthesized in Figure 2.

As conserved heterotrimeric complexes, NF-Ys integrate endogenous developmental signals and exogenous environmental cues to modulate the expression of target genes, acting as key regulators for optimizing horticultural plant growth, yield quality, and stress adaptability. While existing studies have clarified the functional roles of NF-Ys in multiple species and biological processes, there remain unaddressed knowledge gaps—such as incomplete regulatory networks and limited translational application—that hinder a comprehensive understanding of their potential, necessitating further targeted research to provide more solid theoretical support for horticultural crop genetic improvement.

Based on the above research status, future studies should first prioritize deciphering the crosstalk between NF-Ys and other regulatory layers in governing development and stress responses. Current research has preliminarily indicated interactions between NF-Ys and miRNAs, epigenetic modifications, or other transcription factors, but the global regulatory networks—including how these components synergize to fine-tune NF-Y function across different developmental stages and stress types—remain unclear. Employing integrative multi-omics approaches (e.g., combining transcriptomics with epigenomics or protein-protein interactomics) to systematically map these networks will help clarify the upstream regulators and downstream effectors of NF-Ys, deepening the understanding of their precise regulatory mechanisms.

Second, investigating NF-Y-mediated mechanisms under combined stress conditions is essential to bridge the gap between laboratory research and field applications. Most current studies focus on single stresses (e.g., drought, hot, cadmium stress), yet horticultural plants in natural environments frequently face concurrent stresses (e.g., drought and temperature stress) [96,97]. Future work should simulate field-relevant combined stress scenarios to explore how NF-Ys coordinate responses—such as whether they mediate cross-tolerance via shared signaling pathways (e.g., ABA signaling) or activate stress-specific cascades. This research will provide direct theoretical support for breeding climate-resilient horticultural cultivars capable of withstanding complex environmental challenges.

Third, efforts should be made to accelerate the functional validation of uncharacterized *NF-Y* genes, especially in understudied specialty horticultural species—such as ornamental flowers (e.g., orchid) and medicinal herbs (e.g., ginseng)—for which only genome-wide gene identification has been reported to date with functional characterization still lagging behind, given that many *NF-Y* genes across horticultural taxa have been identified via genome-wide analysis but lack in-depth functional characterization, and emerging regulatory modes of NF-Ys (e.g., involvement in secondary metabolism or beneficial plant-microbe interactions) also warrant exploration. Applying high-throughput functional screening techniques (e.g., virus-induced gene silencing, CRISPR/Cas9-based gene editing) to these uncharacterized candidates will not only expand the functional repertoire of NF-Y transcription factors but also enrich genetic resources for improving species-specific traits (e.g., ornamental value, medicinal component content) of specialty horticultural plants.

Fourth, translating NF-Y-related basic research into practical breeding tools needs to be strengthened. While some *NF-Y* genes have been validated as promising targets for trait improvement (e.g., enhancing stress tolerance, optimizing flowering time), challenges like off-target effects or pleiotropy (e.g., improved stress resistance trading off with yield) limit their application. Future translational research should focus on precision genetic modification—such as using tissue-specific or stress-inducible promoters to control NF-Y expression—and gene stacking to improve multiple traits simultaneously. Combining laboratory validation with field trials will ensure the stability and practicality of NF-Y-modified cultivars, fully exploiting the potential of NF-Ys for addressing key challenges in horticultural production.

Finally, a critical assessment of the translational challenges is essential to bridge NF-Y research and practical breeding. While the agronomic potential of NF-Ys is clear, several key bottlenecks must be addressed. Genetic redundancy within expanded NF-Y families, especially in polyploid or perennial crops, may buffer the effect of single-gene modifications, necessitating strategies like multiplex gene editing. Pleiotropic effects and trade-offs—where improved stress tolerance might compromise yield or development—require solutions such as the use of tissue-specific or stress-inducible promoters for precise spatiotemporal control. Ultimately, application must be evidence-based and context-specific, moving beyond proof-of-concept in model systems to demonstrate robust, field-validated performance in target horticultural species without adverse agronomic penalties.

## Figures and Tables

**Table 1:** Genomic and functional overview of *NF-Y* gene families in horticultural plants.

Plant Species	Subunit Gene Numbers	Chromosome Distribution Characteristics	Key Functional Roles Studied	Ref(s).
*Brassica campestris* (Flowering Chinese cabbage)	A *: 17, B: 20, C: 12	Uneven; 10 genes on chr * A09, only 1 on chr A04	Bolting and flowering	[26]
*Brassica campestris* (Non-heading Chinese cabbage)	A: 20, B: 24, C: 13	Random on 10 chrs; max (10) on chr9, min (1) on chr4	Flowering	[17]
*Camellia sinensis* (Tea plant)	C: 9	Located on 9 different chrs	Growth, development, abiotic stress	[27]
*Camellia sinensis* (Tea plant)	A: 10, B: 15, C: 10	N/A	Drought stress, ABA signaling, photosynthesis	[28]
*Capsicum annuum* (Pepper)	B: 19	On 7 of 12 chrs; tandem duplication on chr7	Salt stress	[22]
*Capsicum annuum* (Pepper)	A: 10, B: 12, C: 9	Distributed across 12 chrs	Cold stress	[29]
*Chrysanthemum seticuspe* (Chrysanthemum)	A: 8, B: 21, C: 17	Uneven; absent on CsG_LG3 *; concentrated on CsG_LG1, CsG_LG5, CsG_LG8	Drought stress, growth	[30]
*Citrullus lanatus* (Watermelon)	A: 7; B: 10; C: 8 [31] A: 7; B: 8; C: 4 [32] A: 7; B: 10; C: 5 [33]	Uneven on 9–10 chrs; often absent on chr5; more genes on chr2, 10	Abiotic/biotic stress, phytohormone response, growth, seed development	[31,32,33]
*Citrus grandis* (Pummelo)	A: 6, B: 13, C: 5	1–5 genes per chr	Sucrose metabolism	[34]
*Citrus sinensis* (Sweet orange)	A: 6, B: 11, C: 5	On all chrs except chr8; segmental and tandem duplications identified	Drought stress, development	[35]
*Cucumis melo* (Melon)	A: 6, B: 11, C: 8	Unevenly distributed on 10 chrs	Growth, fruit ripening, cold stress	[36]
*Cucumis sativus* (Cucumber)	A: 7, B: 13, C: 7	On all chrs except chr2; max (7 each) on chr3, 6	Fruit development, drought and salt stress	[14]
*Cymbidium sinense* (Cymbidium)	A: 5, B: 9, C: 13	Uneven on 13 chrs; max (5) on chr9, min (1) on chr3, 4, 6, 17	Drought stress	[37]
*Fragaria vesca* (Woodland strawberry)	A: 6, B: 12, C: 5	Uneven on 7 chrs; max on Fvb3 (7), Fvb6 (6); segmental duplication events	Phylogeny, expression profiles, subcellular localization	[21]
*Ginkgo biloba* (Ginkgo)	A: 7, B: 12, C: 6	Random on 11 chrs; none on chr4; segmental duplications	Heat stress	[38]
*Malus domestica* (Apple)	A: 11, B: 22, C: 10	Uneven on 16 chrs (except chr08); 1–4 genes/chr; concentrated on termini of chr03, 04, 05, 10, 11	Growth, abiotic stress	[13]
*Musa acuminata* (Banana)	A: 14, B: 16, C: 14	Uneven on 11 chrs & 1 scaffold; max on chr4/8; expansion via WGD	Fruit ripening regulation	[25]
*Panax ginseng* (Ginseng)	A: 13, B: 14, C: 13	N/A	Phylogeny, expression, salt stress	[39]
*Petunia hybrida* (Petunia)	A: 10, B: 13, C: 4	N/A	Tissue expression, abiotic stress	[40]
*Phalaenopsis* sp. (Butterfly orchid)	A: 4, B: 9, C: 11	Segmental and tandem duplication events	Flowering, cold stress, ABA response	[23]
*Prunus armeniaca* (Apricot)	A: 6, B: 15, C: 7	Uneven on 8 chrs; max on chr4 (8), chr6 (7)	Oil biosynthesis in kernels	[41]
*Prunus mume* (Japanese apricot)	A: 5, B: 13, C: 9	Distributed on 8 chrs; max on Chr01, Chr03 (25.92% each), min on Chr06 (3.70%)	Flower bud dormancy, phytohormone response, cold stress	[24]
*Prunus mume* (Plum blossom)	A: 6, B: 13, C: 8	N/A	Abiotic stress	[42]
*Prunus persica* (Peach)	A: 6, B: 12, C: 6	On all 8 chrs; max (8) on chr4, min (1) on chr8	Drought stress, tissue expression, evolutionary relationships	[43]
*Solanum lycopersicum* (Tomato)	A: 10	Uneven across 7 of 12 chrs (e.g., Chr01:3 genes)	Saline-alkali and drought stress	[44]
*Solanum lycopersicum* (Tomato)	A: 10, B: 29, C: 20	57 genes on 12 chrs; max *NF-YB* on chr5 (7), max *NF-YC* on chr3 (6); no *NF-YA* on chr4–7, 9	Flowering time, embryogenesis, seed maturation, and fruit development	[15,16]
*Solanum tuberosum* (Potato)	A: 11; B: 20; C: 6 [18] A: 10; B: 22; C: 9 [45]	Uneven on 12 chrs; max (7 each) on chr1,5; min (1) on chr8; high density on proximal chr1 and distal chr5	Development, abiotic/biotic stress, anthocyanin biosynthesis	[18,45]
*Vaccinium corymbosum* (Blueberry)	A: 24	Distributed on multiple scaffolds (e.g., S4, S9, S11, S17, S19, S20)	Abiotic stress, tissue expression	[46]
*Vitis vinifera* (Grape)	A: 8, B: 18, C: 8	32 genes on 14 chrs; uneven; max (5 genes each) on chr6, 19	Abiotic/biotic stress, hormone signaling, sugar metabolism, development	[47]
*Zingiber officinale* (Ginger)	A: 10, B: 16, C: 10	Uneven on 11 chrs; high density on chr06, 11; none on chr01	Rhizome and flower development, abiotic stress	[48]
*Ziziphus jujuba* (Jujube)	A: 8, B: 15, C: 9	27 genes on 11 chrs (except chr6), 5 genes on unplaced scaffolds	Abiotic stress, development	[49]

* A: *NF-YA*; B: *NF-YB*; C: *NF-YC*; chr, chromosome; LG, linkage group. Summary: This comparative overview highlights the significant expansion and diversification of the NF-Y family across major horticultural species, primarily driven by whole-genome and tandem duplications. The variation in subunit numbers and their non-random chromosomal distribution underscore lineage-specific evolutionary trajectories. Functionally characterized roles to date are noted, revealing a primary research focus on abiotic stress and reproductive development, while many species await deeper investigation.

**Table 2:** NF-Y transcription factors regulating phase transition and flowering in horticultural plants.

Plant Species	*NF-Y* Gene	Expression Pattern	Molecular Regulatory Mechanisms	Phenotypic Effect	Ref.
*Pyrus bretschneideri* (Chinese white pear)	*NF-YA4a*	↑ * during ontogeny, peaks in adult phase; higher in adult vs. juvenile tissues	→ * flowering transition pathway activation	↑ flowering in transgenic *Arabidopsis*	[56]
*Brassica campestris* (Non-heading Chinese cabbage)	*BcNF-YA8*	↑ in roots; ↑ under ABA	→ *BcFT* ↑; interacts with BcMAX2 * → *BcBRC1* ↑; ↓ AsA * accumulation	↑ flowering in overexpression lines; ↓ * flowering in silenced lines	[17]
*Chrysanthemum indicum* (Wild chrysanthemum)	*CiNF-YB8*	↓ during plant aging	Forms heterotrimer → binds CCAAT box → *cin-MIR156ab* ↑	↑ vegetative transition & ↑ flowering	[57]
*Solanum lycopersicum* (Tomato)	*SlNF-YA3b*	↓ in flower buds/flowers	Binds CCAAT motif of *SFT* promoter → *SFT* ↓	↑ flowering in knockout lines	[54]
*Solanum lycopersicum* (Tomato)	*SlNF-YA8*	Expressed in seedlings, stems, inflorescences, and fruits; ↓ in floral buds of early-flowering mutants	ZFN-mediated disruption of DNA-binding domain regulates flowering time-related genes (e.g., *FT* homologs)	15% of mutant lines show early flowering; altered inflorescence architecture (increased florets per inflorescence)	[58]
*Malus domestica* (Apple)	*MdNF-YB18*	Higher in ‘Qinguan’; peaks at MS * stage	Interacts with MdNF-YC3/7 → binds *MdFT1* promoter → *MdFT1*↑; interacts with MdCOLs *	↑ flowering in transgenic *Arabidopsis*	[55]
*Petunia hybrida* (Petunia)	*PhNF-YC2*	↑ in apical buds/leaves before flowering	→ PhCO/PhGI/PhFBP21/PhGA20ox4/PhSPL9b * ↓	↓ flowering & ↓ chlorophyll content	[59]
*Petunia hybrida* (Petunia)	*PhNF-YC4*	↑ in roots/leaves/buds before flowering	→ *PhCO/PhGI/PhFBP21/PhGA20ox4/PhSPL9b*↓	↓ flowering	[59]
*Lycoris chinensis* (Lycoris)	*NF-YB3(c105794g3)*	↑ in flowering bulbs (P5)	→ FT/SOC1 * ↑	Putative ↑ floral transition	[60]
*Juglans regia* (Walnut)	*JrNF-YB4/* *Y* *6/YC1/* *YC* *3/* *YC* *7*	↑ in leaf/female flower buds (FB-4)	Bind *JrFT* promoter → *JrFT* ↑; JrCO-JrNF-YB-JrNF-YC complex → *JrFT* transcription ↑	↑ female flower differentiation and flowering	[61]
*Prunus mume* (Japanese apricot)	*PmNF-YA3/YB3/YC1*	↓ during dormancy; ↑ during release	miR169 → *PmNF-YA3* ↓; GA4 → PmRGL2 * ↓ → NF-Y complex activation	↑ bud sprouting	[62]
*Brassica juncea* (Chinese mustard)	*BjuNF-Y*	↑ in LF/MF2 subgenomes during floral transition	NF-Y-CO complex → *SOC1* ↑	Altered flowering time	[63]
*Chrysanthemum seticuspe* (Chrysanthemum)	*CmNF-YB8*	↓ during development	Binds *cmo-MIR156* promoter → *cmo-MIR156* ↑ → *SPL3/5/9* ↓	↑ flowering under LD/SD *	[64]
*Carya illinoensis* (Pecan)	*NF-YA1*	↑ in early male bud development	→ vegetative-to-reproductive transition↑	Facilitates ↑ male flower bud differentiation and development	[65]
*Lilium spp.* (Lily)	*LoNFYA7*	↑ in dormant central buds; ↓ in growth-transited bulbs	LoNFYA7-LoVIL1-PRC2 complex → *LoCALS3* ↓ (via H3K27me3) → *LoFT1*↑	↑ central bud growth	[19]
*Brassica campestris* (Flowering Chinese cabbage)	*BcNF-YA8/YB14/YB20/YC5*	↑ during stalk development/flowering	Interacts with BcRGA1 * → GA signaling regulation	Potential regulators of ↑ bolting/flowering	[26]
*Phalaenopsis* sp. (Butterfly orchid)	*PhNF-YA1/* *Y* *A3/YB6/YC7*	↑ in vegetative/inflorescence buds; ↑ under low-temperature	Complex → *PhFT3* ↑ & PhSVP * ↓	↑ floral transition	[23]
*Rosa hybrida* (Rose)	*RhNF-YC9*	↑ Early flower opening (peaked at stage 2), then declined; ↓ by ethylene	↑ *RhGA20ox*/↓ *RhGA2ox* & ↑ 11 cell expansion genes	Silencing: ↓ petal ^^a^ expansion, size & AbsE * cell size	[66]

* →: regulatory relationship/putative involvement; ↑: upregulation/activation/promotion; ↓: downregulation/repression/suppression; MAX: MORE AXILLARY GROWTH; BRC: BRANCHED; AsA: Ascorbic Acid; MS: middle stage of flower bud differentiation; CO: CONSTANS; COL: CO-LIKE; GI: GIGANTEA; FBP: FLOWER BINDING PROTEIN; GA20ox: GA20 oxidase; SPL: SQUAMOSA PROMOTER BINDING PROTEIN-LIKE; SOC: SUPPRESSOR OF OVEREXPRESSION OF CO; RGL: REPRESSOR OF GA1 LIKE; LD: long day; SD: short day; RGA: REPRESSOR OF *ga1-3*; SVP: SHORT VEGETATIVE PHASE; AbsE: abaxial sub-epidermal. ^^a^: petal refers to the corolla component of rose floral envelopes, which consists of calyx and corolla. Summary: Collectively, these studies position NF-Y complexes as versatile, context-dependent integrators of flowering signals. Their dual role as both promoters and repressors can be attributed to three factors: (1) subunit specificity of the NF-YA/B/C trimer; (2) integration of opposing hormonal cues (e.g., ABA vs. GA); and (3) regulatory node within the network (direct target vs. upstream modulator). A conserved theme is their interaction with core flowering pathways (FT/SOC1/CO), often mediated by hormonal or epigenetic cross-talk.

**Table 3:** NF-Y transcription factors regulating early embryogenesis, organ morphogenesis, and seed maturation with storage compound accumulation in horticultural plants.

Plant Species	*NF-Y* Gene	Expression Pattern	Molecular Regulatory Mechanisms	Phenotypic Effect	Ref.
*Prunus armeniaca* (Apricot)	*PaNF-YA2/* *Y* *A6*	↑ * in kernels across stages	Positively correlated with oil content; → fatty acid metabolism and oil accumulation	↑ oil biosynthesis in kernels	[41]
*Prunus armeniaca* (Apricot)	*PaNF-YB4*	↑ in kernels	Homologous to *AtLEC1* *; → * ↑ fatty acid synthesis genes	↑ oil content in kernels	[41]
*Musa acuminata* (Banana)	*MaNF-YA5/YB1/YB2/YC9/YC11/YC14*	↑ during ripening; ethylene ↑, 1-MCP ↓ *	Form heterotrimers to act as transcriptional activators; → ethylene signaling	↑ fruit ripening (e.g., peel color change)	[25]
*Musa acuminata* (Banana)	*MaNF-YA1/YA3/YA6/YB3/YB6/YC2/YC5*	↓ during ripening; ethylene ↓, 1-MCP ↑	Function as transcriptional repressors; form inhibitory complexes	↓ fruit ripening (e.g., delayed under 1-MCP)	[25]
*Phaseolus vulgaris* (Common bean)	*NF-YA1/YA9*	↓ at 10 DAA *	↓ by pvu-miR169k	Involved in defining embryogenesis time course	[70]
*Phaseolus vulgaris* (Common bean)	*LEC1* (*NF-YB9*), *L1L* (*NF-YB6*)	Expressed during seed filling	Act as master regulators of seed filling, modulated by MIR169-NF-YA	Control legume seed filling process	[70]
*Cucumis sativus* (Cucumber)	*CsNF-YC2/YC9*	↑ by light	Binds *CsTIC21* promoter to ↑ transcription → chloroplast photomorphogenesis	Silencing → etiolated growth and ↓ chlorophyll content	[68]
*Solanum tuberosum* (Potato)	*StNF-YA8*	↑ during tuber dormancy release	Forms module with StNF-YB20 and StNF-YC5 to ↑ transcription of GA and ABA pathway genes	↑ tuber dormancy release; overexpression ↑ sprouting	[69]
*Solanum tuberosum* (Potato)	*StNF-YC4*	↑ in shoot apex, stem, roots, tubers	Promoter has AGAMOUS element; → flowering time regulation	↑ tuber protein content; no impact on yield or appearance	[71]
*Citrus grandis* (Pummelo)	*CgNF-YB9*	V-shaped expression in juices sacs (minimum at 120 DAF *, ↑ at 180/240 DAF)	↓ sucrose-phosphate synthase genes; ↑ vacuolar invertase genes → sucrose conversion	↓ sucrose content; ↑ fructose and glucose in transgenic tobacco	[34]
*Carthamus tinctorius* (Safflower)	*CtNF-YB12*	↑ during early seed development	↑ genes for fatty acid biosynthesis and glycolysis	Heterologous expression ↑ seed pod length, size, oil content, and alters fatty acids	[72]
*Fragaria ananassa* (Strawberry)	*YZ9* (*XM_004291519.2*)	↑ fruit coloring	Regulates anthocyanin and cell wall genes; interacts with CBF */NF-Y pathway	↑ fruit coloring under cold; ↑ pectin and cellulose; ↑ linalool aroma	[73]
*Solanum lycopersicum* (Tomato)	*SlNF-YA8*	Expressed in fruits, cotyledons, and stems; ↑ in developing fruits	ZFN-mediated mutation of DNA-binding domain regulates cell division/expansion genes and fruit shape-related pathways	Mutants show ↑ fruit weight, rounded fruit shape, altered locule number, and abnormal cotyledon development	[58]
*Solanum lycopersicum* (Tomato)	*SlLEC1-LIKE4* (*SlL1L4*)	Expressed in flowers, immature green fruits, seeds, and leaves; ↑ during fruit ripening	ZFN-mediated disruption → alters fruit metabolic pathways (sugar/organic acid metabolism) and seed maturation-related genes	Mutants show altered fruit composition (↑ fructose, ↓ oxalate), ↓ ripening, and abnormal embryogenesis	[67]
*Solanum lycopersicum* (Tomato)	*SlNF-YA3b*	↑ during fruit ripening	Binds *SlPDS* * promoter to ↑ transcription	Silencing → ↓ carotenoid accumulation and abnormal coloration	[74]
*Solanum lycopersicum* (Tomato)	*NF-YB8a/b/c*, *NF-YC1a/b/d*, *NF-YC9/YA1b/YA9*	↑ in fruit peels during ripening	Forms NF-Y complex to bind CCAAT in *CHS1* * promoter; modulates H3K27me3; cooperates with MYB12 * → ↓ flavonoid genes	Leads to pink fruits with colorless peels due to ↓ naringenin chalcone	[75]
*Citrullus lanatus* (Watermelon)	*ClNF-YB9*	Seed-specific expression; peaks at 20 DAP * and 45–50 DAP	Interacts with ClNF-YCs to form heterodimers; recruits ClNF-YA7; binds CCAAT motifs	Knockout → abnormal leaf cotyledon and seed abortion; 43% seeds show no dormancy	[33]
*Fragaria vesca* (Woodland strawberry)	*FveNF-YB3*	↑ in achenes tissues	Homologous to *AtNF-YB6*; targeted by miR395	Contributes to seed maturation	[21]

* →: regulatory relationship/putative involvement; ↑: upregulation/activation/promotion; ↓: downregulation/repression/suppression; LEC1: LEAFY COTYLEDON 1; 1-MCP: 1-methylcyclopropene; DAA: days after anthesis; DAF: days after flowering; CBF: C-REPEAT BINDING FACTOR; PDS: PHYTOENE DESATURASE; CHS: CHALCONE SYNTHASE; MYB: MYB DOMAIN PROTEIN; DAP: days after pollination. Summary: NF-Y regulation extends from embryogenesis to the maturation of horticulturally vital organs. A conserved module involves LEC1-type NF-YBs master-regulating seed filling and storage compound accumulation. In fruits and tubers, specific NF-Y trimers interface with hormone (ethylene/ABA) and sugar signaling to control ripening and dormancy. This illustrates how an ancestral developmental regulator has been co-opted for specialized adaptation in horticultural species, directing resource allocation to harvested organs.

**Table 4:** NF-Y transcription factors involved in drought stress responses in horticultural plants.

Plant Species	*NF-Y* Gene	Induction Conditions (Drought)	Molecular Regulatory Mechanisms	Resistant Phenotype	Ref.
*Amaranthus hypochondriacus* (Amaranth)	*AhNF-YC*	Withhold irrigation for 4/6/8 d	ABA-dependent pathway	↑ * water deficit tolerance	[79]
*Malus hupehensis* (Apple)	*MhNF-YA2*	10% PEG *-6000, 0–24 h; Water deprivation for 30 d	Binds *MhHSP70-3* * promoter ↑	↑ drought tolerance; ↓ * ROS, MDA *, water loss	[80]
*Malus hupehensis* (Apple)	*MhNF-YA3-like*	Soil drought for apple plants: withhold watering 8–12 d; 100 μM ABA for apple calli 20 d; 400 mM mannitol (apple 3 d)/100 mM (calli 20 d)	Interacts with MhMSI4-like, activates *MhAAO3* for ABA synthesis	↑ drought tolerance; ↓ ROS and membrane damage	[76]
*Malus domestica* (Apple)	*MdNF-YC5/8*	10% PEG-6000 treatment	Interacts with MdNF-YBs; promoter MBS *	↑ drought resistance (root development)	[13]
*Chrysanthemum seticuspe* (Chrysanthemum)	*CmNF-YB8*	Withholding water for 30 d; Dehydration (air exposure, 23 ± 1 °C)	Regulates *CmCIPK6/CmSHN3* → * stomatal movement	*RNAi*: ↑ drought resistance	[77]
*Citrus spp.* (Citrus)	*CiNF-YA1*	Natural drought (5 stages); 100 mM mannitol (callus)	Activates CiFT with CiNF-YB2/YC2	Silencing: ↑ drought tolerance	[78]
*Cymbidium sinense*	*CsNF-YBs*	Mild drought: 3 d of water withholding; Severe drought: 7 d of water withholding	Promoter MBS motifs; interacts with DR1/BZIP *	↑ drought tolerance (potential)	[37]
*Zingiber officinale* (Ginger)	*ZoNF-YB8*	15% PEG-6000, 1/3/6/12/24/48 h	Binds CCAAT boxes; ↑ stress genes	↑ multi-stress tolerance	[48]
*Zingiber officinale* (Ginger)	*ZoNF-YB7/16*	15% PEG-6000, 1/3/6/12/24/48 h	ABA-mediated via ABRE *	↑ drought tolerance (stomatal closure)	[48]
*Vitis amurensis* (Grapevine)	*VaNF-YA6,* etc.	10% PEG-6000 for 24 h	Forms heterotrimers; interacts with SOS2/ABF3 *	↑ salt/drought tolerance	[81]
*Vitis amurensis* (Grapevine)	*VaNF-YA6*	Natural drought for 15 d	↑ *SOS2/SOS3/ABF3*; ↑ antioxidants, proline, ABA	↑ Fv/Fm; ↓ electrolyte leakage	[81]
*Ziziphus jujuba* (Jujube)	*ZjNF-Ys*	10% PEG-6000 for 3 h	Promoter MYB/MYC * cis-elements	↑ drought/salt tolerance (candidate)	[49]
*Cajanus cajan* (Pigeon pea)	*NF-YA7*, *NF-YA10*	Water withholding (11 d)	ABA-dependent; ↑ *PYR1* * expression	↑ RWC *; ↓ MDA; ↑ drought adaptation	[82]
*Prunus mume* (Plum blossom)	*PmNF-Ys*	300 mM mannitol for 3/6/12/24 h	Binds CCAAT; ↑ stress genes	N/A	[42]
*Solanum tuberosum* (Potato)	*StNF-YA3*	Water withholding (7 d)	Repressed by StmiR169a (↓); ↑ ROS scavenging genes	↑ drought resistance; ↑ photosynthesis	[83]
*Solanum tuberosum* (Potato)	*StNF-YA7*	15% PEG-6000 treatment on potato seedlings	Regulates drought-resistance genes	↑ drought tolerance	[84]
*Solanum tuberosum* (Potato)	*StNF-YA7*	Water withholding for 3 weeks	↑ *StWRKY75* *; ↓ *StMYB102/ANAC038* *	↑ drought tolerance; ↑ survival rate	[85]
*Solanum tuberosum* (Potato)	*StNF-YA7.2*	Drying on Whatman 3MM paper (1/3 h)	Induced via miR169 (↓)	↑ water deficit tolerance	[18]
*Solanum tuberosum* (Potato)	*StNF-YA9.1/9.2*	Drying on Whatman 3MM paper (1/3 h)	Activates ABA signaling; ↑ *LEA* * genes	↑ drought/salt tolerance; ↓ water loss	[18]
*Solanum tuberosum* (Potato)	*StNF-YC4*, *YA3/5*	Withhold irrigation for 4/9/21/17 d	Regulates ABA-responsive genes, *HSP*s	↑ drought tolerance	[86]
*Solanum tuberosum* (Potato)	*StNF-YC9*	20% PEG-6000 irrigation (300 mL/pot); Soil RWC = 45% for 7/14 d; Detached leaves air-drying (24 °C)	↑ stomatal closure; ↑ antioxidants, proline	↑ RWC; ↑ photosynthesis; ↓ MDA	[87]
*Citrus sinensis* (Sweet orange)	*CsNF-YA5*	Leaf predawn Ψ * = −1.5 MPa	↓ H_2_O_2_; ↑ antioxidants, photosynthesis	↑ biomass; ↑ root length; ↓ dehydration rate	[35]
*Camellia sinensis* (Tea plant)	*CsNF-YC6*	15% PEG-6000 for 7 d; 100 mM mannitol (callus)	↑ ABA signaling genes; ↑ proline	↑ drought tolerance	[27]
*Camellia sinensis* (Tea plant)	*CsNF-YB3*, *CsNF-YC2*	10% PEG-6000, 0/6/24/48 h	No drought-responsive cis-elements	↓ drought tolerance	[28]

* →: putative involvement; ↑: upregulation/activation/enhancement; ↓: downregulation/repression/reduction; PEG: polyethylene glycol; HSP: HEAT SHOCK PROTEIN; MDA: malondialdehyde; MBS: MYB Binding Site; DR1: DOWN-REGULATOR OF TRANSCRIPTION 1; BZIP: BASIC LEUCINE ZIPPER; ABRE: ABA-responsive element; SOS: SALT OVERLY SENSITIVE; ABF: ABA-RESPONSIVE ELEMENT-BINDING FACTOR; MYC: MYELOCYTOMATOSIS; PYR1: PYRABACTIN RESISTANCE 1; RWC: relative water content; WRKY: WRKY domain-containing TF; ANAC: *Arabidopsis* NAC domain-containing protein; LEA: LATE EMBRYOGENESIS ABUNDANT; Ψ: water potential. Summary: NF-Y-mediated drought tolerance predominantly converges on two core mechanisms: (1) the amplification of ABA biosynthesis and signaling, leading to stomatal closure and stress-responsive gene expression; and (2) the enhancement of ROS scavenging capacity. While this ABA-centric module is widely conserved, species-specific adaptations exist, such as modulating cuticular wax (chrysanthemum). The evidence landscape ranges from validated regulatory modules to correlative expression associations, highlighting priorities for functional validation.

**Table 5:** NF-Y transcription factors involved in salt stress responses in horticultural plants.

Plant Species	*NF-Y* Gene	Induction Conditions (Salt)	Molecular Regulatory Mechanisms	Resistant Phenotype	Ref.
*Pyrus bretschneideri* (Chinese white pear)	*PbrNF-YA4a*	0/50/100 mM NaCl (10 d)	OE * enhances salt tolerance via unknown downstream pathways	↑ * Germination & green cotyledons under salt (Transgenic *Arabidopsis*)	[56]
*Vaccinium corymbosum* (Blueberry)	*VcNF-YA1–A24*	200 mM NaCl (0–24 h)	Bind stress-responsive cis-elements	↑ Abiotic stress adaptation (Expression data)	[46]
*Vitis amurensis* [Grapevine (wild)]	*VaNF-YA6*, *VaNF-YB5*, etc.	200 mM NaCl (24 h)	Form heterotrimers; putatively interact with SOS/ABF/CPK * pathways	↑ Salt tolerance candidate (Expression data)	[81]
*Vitis amurensis* [Grapevine (cultivated)]	*VaNF-YA6*	300 mM NaCl (24 h for RT-qPCR and physiological index determination, 48 h for Fv/Fm test)	↑ *SOS2/3*, *ABF3*, *CPK6*; ↑ antioxidants, proline, ABA; ↓ * H_2_O_2_, MDA	↑ Fv/Fm; ↓ electrolyte leakage (Transient transformation of grapevine leaves)	[81]
*Solanum lycopersicum* (Tomato)	*SlNF-YA10a*	300 mM saline-alkali (5 d, pH 8.90)	↓ SOD/CAT * activities; disrupts ion homeostasis	↑ Electrolyte leakage & MDA (OE); ↑ tolerance (KO *)	[44]
*Solanum lycopersicum* (Tomato)	*SlNF-YC1*	300 mM saline-alkali (4 d, pH 8.90)	Interacts with SlMYB1; ↑ *SlGAD1* → * GABA accumulation; activates ethylene signaling & ROS scavenging	*OE*: ↑ saline-alkali tolerance (↓ ion leakage, MDA, H_2_O_2_; ↑ GABA & ethylene); *RNAi*: ↑ saline-alkali sensitivity	[88]
*Camellia sinensis* (Tea plant)	*CsNF-YC6*	200 mM NaCl (Arabidopsis, 7 d)	Activates ABA pathway genes (*ABI5* *, *NCED3* *); ↑ proline; ↓ MDA	↑ Germination & root length under salt (Transgenic *Arabidopsis*)	[27]
*Capsicum annuum* (Pepper)	*CaNFYB01*, *18*, *19*	200 mM NaCl (12/24 h)	Promoter contains ABRE, MeJA *-responsive elements	↑ Early salt response (Expression data)	[22]
*Solanum tuberosum* (Potato)	*StNF-YB5.1*	200 mM NaCl (3/12 h)	Regulates ion and osmotic homeostasis	↑ Salt tolerance (Expression data)	[18]
*Ziziphus jujuba* (Jujube)	*ZjNF-YA2/4/7/8*, *YB3/5/6/13/14/15*, *YC3/4*	150 mM NaCl (3 h)	Promoter contains MYB/MYC/DRE * stress-responsive elements	↑ salt tolerance candidate (Expression data)	[49]
*Malus domestica* (Apple)	*MdNF-YB3*	100 mM NaCl, 0/6/12/24 h	Promoter contains LTRE *; expression peaks at 24 h	↑ Cold/salt adaptation (Expression data)	[13]
*Cucumis sativus* (Cucumber)	*CsNF-YA6*	200 mM NaCl, 0/6/12/24 h	Key regulator in salt response (23.2-fold change)	↑ Salt tolerance (Expression data)	[14]
*Citrullus lanatus* (Watermelon)	*ClNF-YA5*	200 mM NaCl (0/3/6/9/12/24 h)	MBS/HSE * elements; potential miR169 regulation	↑ Salt tolerance (putative, Expression data)	[32]
*Raphanus sativus* (Radish)	*RsNF-YA2* */* *3*	200 mM NaCl (3/6/12/24/48/96 h)	Activated via ↓miR169; regulate downstream stress genes & ion homeostasis	↑ Salt adaptation (Expression & miRNA-target regulation)	[89]

* →: regulatory relationship/putative involvement; ↑: upregulation/activation/enhancement; ↓: downregulation/repression/reduction; OE: overexpression; CPK: CALCIUM-DEPENDENT PROTEIN KINASE; SOD: superoxide dismutase; CAT: catalase; KO: knockout; ABI: ABA INSENSITIVE; NCED: 9-cis-epoxy carotenoid dioxygenase; MeJA: methyl jasmonate; DRE: dehydration-responsive element; LTRE: low-temperature responsive element; HSE: heat shock element. Summary: NF-Ys enhance salt tolerance through intertwined strategies of ionic homeostasis (e.g., regulating SOS pathway genes) and osmotic adjustment (e.g., promoting proline or GABA accumulation). A key regulatory layer involves the miR169-NF-YA module, where stress-induced miR169 downregulation de-represses specific NF-YA targets. The compiled studies reveal a focus on early signaling responses, with functional validation often needed to delineate precise downstream targets for ion transport versus osmotic protection.

**Table 6:** NF-Y transcription factors involved in heat stress responses in horticultural plants.

Plant Species	*NF-Y* Gene	Induction Conditions (Heat)	Molecular Regulatory Mechanisms	Resistant Phenotype	Ref.
*Malus domestica* (Apple)	*MdNF-YA3*	40 °C, 0/6/12/24 h	Binds ABRE; interacts with TFs → ↑ * stress genes	↑ heat tolerance	[13]
*Brassica oleracea* (Cabbage)	*BoDPB3-1/NF-YC10*	42 °C for 3 h and 5 h	Trimer with NF-YA2/B3 → * activates *DREB2A*	↑ Thermotolerance in tolerant cultivars	[91]
*Cucumis sativus* (Cucumber)	*CsNFYA1*	43 °C (16 h day/8 h night)	Interacts with CsMBF1c → ↑ heat-related genes	OE in *Arabidopsis* → ↑ thermotolerance	[90]
*Zingiber officinale* (Ginger)	*ZoNF-YB5*	40 °C, 0/1/3/6/12/24 h	Negative regulator of stress signaling	Potential role in acclimation	[48]
*Ginkgo biloba* (Ginkgo)	*GbNF-YA6*	40 °C, 0/1/3/6 h	Interacts with GbHSP; ↑ *HSF*s expression	OE in *Arabidopsis* → ↑ survival under HS	[38]
*Vitis vinifera* (Grape)	*VvNF-YB9*	45 °C heat, then 25 °C recovery	Involved in heat stress recovery in grape berries	Role in grape berry thermotolerance	[47]
*Petunia hybrida* (Petunia)	*PhNF-YA1/2/10*	40 °C, 0/1/3/6/12 h	Rapid response (1 h); *PhNF-YA7* ↓ * at 6 h	Putative role in heat tolerance	[40]
*Solanum lycopersicum* (Tomato)	*SlNF-YA9/10*	45 °C for 4.5 h	miR169 cleavage → represses *HSFA3/7*	↑ Thermotolerance, photosynthesis, WUE *	[20]

* →: putative involvement; ↑: upregulation/enhancement; ↓: downregulation/repression; WUE: water use efficiency.

**Table 7:** NF-Y transcription factors involved in cold stress responses in horticultural plants.

Plant Species	*NF-Y* Gene	Induction Conditions (Cold)	Molecular Regulatory Mechanisms	Resistant Phenotype	Ref.
*Vaccinium corymbosum* (Blueberry)	*VcNF-YA1–A24*	4 °C, 3 h	Binds stress-responsive cis-elements; regulates downstream genes	↑ * abiotic stress tolerance	[46]
*Zingiber officinale* (Ginger)	*ZoNF-YA7*, *ZoNF-YC7*	4 °C, 0/1/3/6/12/24/48 h	Binds LTRE; activates cold-responsive genes	↑ cold tolerance; cellular homeostasis	[48]
*Fragaria ananassa* (Strawberry)	*YZ9* (*XM_004291519.2*)	4 °C, 5/7 d	Activates *CBF*; ↑ ROS scavenging; modulates ABA/JA	↑ cold tolerance; ↓ * chilling injury	[73]
*Petunia hybrida* (Petunia)	*PhNF-YA4/5/6/10*, *PhNF-YB13*	4 °C, 1/3/6/12 h	Divergent expression; potential hot/cold overlap	Involved in cold response	[40]
*Cucumis melo* (Melon)	*CmNF-YA1*, *B6/10*, *C1/2/5/7/8*	15 °C (day)/6 °C (night), 0/6/12/18/24 h	Upregulated throughout cold treatment; regulates gene expression	↑ cold tolerance	[36]
*Capsicum annuum* (Pepper)	*CaNF-YC1*	4 °C, 1/3/6/12/24 h	Activates *CaCBF1a/b*, interacts with CaTIFY7, upregulates cold-responsive genes	↑ cold tolerance when overexpressed	[29]

* ↑: upregulation/activation/enhancement; ↓: downregulation/repression/reduction. Summary: NF-Ys occupy key positions in thermotolerance networks, often acting as negative regulators that are suppressed under stress (e.g., via miR169) to de-repress heat shock factors (HSFs) or CBFs. Alternatively, some act as co-activators (e.g., with DREB2A or MBF1c). This duality underscores their role as tunable switches within stress-response circuits. Cold responses frequently involve NF-Y interaction with CBF/DREB1 hubs, linking cold perception to antioxidant and osmotic adjustments.

**Table 8:** NF-Y transcription factors involved in additional stress responses in horticultural plants.

Plant Species	Stress Type	*NF-Y* Gene	Induction Conditions	Molecular Regulatory Mechanisms	Resistant Phenotype	Ref.
*Solanum lycopersicum* (Tomato)	Oxidative stress	*SlNFYA10*	MV * spray, 3 d	Binds *SlGME1*/*GGP1* promoters ↓ * their expression ↓ AsA	OE: ↑ * oxidative stress sensitivity	[92]
*Camellia sinensis* (Tea plant)	Nutrient stress	*NF-Y* family	N/P/K * starvation (hydroponic), 30 d	Putatively regulates biosynthesis genes via CCAAT box	↑ adaptation to nutrient starvation	[95]
*Cajanus cajan* (Pigeon pea)	Aluminum stress	*CcNFYB3*	1 mM AlCl_3_, 3 d	Binds *CcMATE35* promoter ↑ *CcMATE35* → * citrate efflux	OE: ↑ Al tolerance (↑ root elongation & citrate efflux, ↓ root tip cell death & callose); RNAi: ↑ Al sensitivity	[93]
*Citrullus lanatus* (Watermelon)	Pathogen stress	*ClNF-YB8*	*B. cinerea* * (2 × 10^5^ spores/mL) inoculation in *Arabidopsis OE* plants	↑ defense genes (e.g., *AtPR1* *, *AtPR5*)	↑ resistance, ↓ necrotic lesions	[31]
*Citrullus lanatus* (Watermelon)	Pathogen stress	*ClNF-YA2*, *YC2*	*B. cinerea* (2 × 10^5^ spores/mL) inoculation in *Arabidopsis OE* plants	↓ defense genes (e.g., *AtPR1*, *AtPR5*)	↓ resistance, ↑ necrotic lesions	[31]
*Citrullus lanatus* (Watermelon)	Pathogen stress	*ClNF-YA3*, *YB1*, *YC4*	*Pst* * DC3000 (OD_600_ = 0.002) inoculation in *Arabidopsis OE* plants	↑ defense genes (e.g., *AtPR1*, *AtPR5*)	↑ resistance, ↓ bacterial growth	[31]
*Phaseolus vulgaris* (Common bean)	Pathogen stress	*Phvul.010G133300* (*NF-YA3*)	*E. diffusa* * (20 conidia/mm^2^), 7 dpi *	Associated with PR * and HR * to powdery mildew	↓ disease severity, ↑ resistance	[94]

* →: regulatory relationship/putative involvement; ↑: upregulation/activation/enhancement; ↓: downregulation/repression/reduction; MV: methyl viologen; N/P/K: Nitrogen/ Phosphorus/ Potassium; *B. cinerea*: *Botrytis cinerea*; *AtPR*: *Arabidopsis thaliana* pathogenesis-related gene; *Pst*: *Pseudomonas syringae* pv. *tomato*; *E. diffusa*: *Erysiphe diffusa*; dpi: days post-inoculation; PR: partial resistance; HR: hypersensitive response. Summary: Beyond major abiotic stresses, NF-Ys function in niche adaptation, such as aluminum tolerance through citrate efflux regulation and pathogen defense via hormone-mediated signaling. Their role in oxidative stress often involves modulating antioxidant biosynthesis pathways (e.g., ascorbate). These diverse functions illustrate the pleiotropic potential of NF-Y complexes and highlight emerging areas—like nutrient stress and plant-microbe interactions—where their regulatory roles are still being uncovered. Collectively, studies across abiotic and biotic stresses underscore that NF-Y transcription factors frequently act as nodal points where hormone signaling pathways (e.g., ABA, JA, SA, ethylene) converge to rewire gene expression for tailored stress adaptation.

## Data Availability

No new data were created or analyzed in this study. Data sharing is not applicable to this article.
